# The comparison of acute toxicity in 2 treatment courses: Three-weekly and weekly cisplatin treatment administered with radiotherapy in patients with head and neck squamous cell carcinoma

**DOI:** 10.1097/MD.0000000000009151

**Published:** 2017-12-22

**Authors:** Jacek Mackiewicz, Agnieszka Rybarczyk-Kasiuchnicz, Izabela Łasińska, Małgorzata Mazur-Roszak, Daria Świniuch, Michał Michalak, Joanna Kaźmierska, Adam Studniarek, Łukasz Krokowicz, Tomasz Bajon

**Affiliations:** a Department of Medical and Experimental Oncology, Heliodor Swiecicki Clinical, Hospital, Poznan University of Medical Sciences, Poland; b Department of Biology and Environmental Studies, University of Medical Sciences, Poznan, Poland; c Department of Diagnostics and Cancer Immunology, Greater Poland Cancer Centre, Poznan, Poland; d Department of Medical Oncology, Malgorzata Medical Center, Srem; e Department of Computer Sciences and Statistics Poznan University of Medical Sciences; f Radiotherapy Department II Greater Poland Cancer Center; g Electroradiology Department, University of Medical Sciences, Poznan, Poland; h Department of General Surgery Rutgers New Jersey Medical School, Newark, New Jersey, USA; i Department of General, Endocrinological Surgery and Gastroenterological Oncology, Poznan University of Medical Sciences, Poznan, Poland.

**Keywords:** chemoradiotherapy, cisplatin, head and neck cancer

## Abstract

The most appropriate cisplatin treatment schedule delivered with radiotherapy in patients with head and neck squamous cell carcinoma (HNSCC) is unknown. The aim of this study was to compare the acute toxicity and its impact on the course of the treatment, administered cisplatin and radiation doses, the length of hospitalization and supportive drugs administration in patients with HNSCC receiving 2 different cisplatin treatment schedules administered with radiotherapy.

In this retrospective analysis, 104 patients with HNSCC were enrolled. Patients received radiation concurrently with 100 mg/m^2^ cisplatin administered 3-weekly (n = 50; group A) or 35 to 40 mg/m^2^ cisplatin administered weekly (n = 54; group B). Chemoradiotherapy was performed in locally and/or regionally advanced disease (stage III–IV), in a definitive radical upfront setting (71.1%) or after surgical resection in patients with high-risk factors (28.8%).

Both study groups were equally distributed in terms of age, gender, stage of the disease, Eastern Cooperative Oncology Group performance score, chronic diseases and primary tumor site. The schedule of cisplatin dosing did not influence the duration of hospitalization, the number of additional supportive drugs (antibiotics, opioids) administered or total doses of received radiotherapy. However, postponement of radiotherapy due to adverse events was significantly more frequent in patients treated with 35/40 mg/m^2^ (55.56% vs 32%; *P* = .015). Furthermore, patients treated with weekly treatment schedule received lower total cisplatin dose (160 mg/m^2^) in comparison to those treated with the 3-weekly schedule (200 mg/m^2^). Grade 3 and 4 mucositis occurred more frequently in patients treated in group A (70% vs 50%; *P = *.037). Leukopenia was also observed more frequently in group A (88% vs 72.2%; *P = *.04), however there was no difference in grade 3/4 leukopenia between both study arms. There was no statistically significant difference in any other adverse effects.

These results do not demonstrate the advantage of modified weekly schedule over standard 3-weekly cisplatin treatment plan. However, severe mucositis occurred more frequently in patients receiving 3-weekly cisplatin, both chemotherapy schedules seemed to present similar toxicity. Due to conflicting efficacy and toxicity, the results and compliance of weekly and 3-weekly cisplatin schedules should be evaluated in further randomized, controlled trials and retrospective studies.

## 1 Introduction

Head and neck squamous cell carcinoma (HNSCC) represent little more than 5% of all registered malignant tumors in Poland (about 7% among men and 1% among women). In the last years there have been about 6600 new cases and 4200 yearly deaths from HNSCC in Poland.^[1]^

According to 2 meta-analysis concurrent chemoradiotherapy (CRT) for HNSCC improves local tumor control and overall survival.^[2,3]^ The addition of chemotherapy to radiotherapy resulted in 6.5% survival benefit in 5 years in patients with HNSCC. ^[2]^ Concurrent CRT is the standard treatment for locally and/or regionally advanced (stage III–IV) HNSCC, in a definitive radical upfront setting or after surgical resection in patients with high-risk features in the pathology specimen.^[4]^

Cisplatin administered at a dose of 100 mg/m^2^ every 3 weeks is the most commonly used chemotherapy schedule in combination with radiotherapy due to highest evidence of clinical benefit. However, only 50% to 60% of patients were able to receive complete 3 planned cycles of 100 mg/m^2^ cisplatin, because of poor tolerability and severe adverse effects.^[5,6]^ In order to reduce the toxicity and improve treatment compliance, alternative schedules including low-dose cisplatin have been used.^[7–11]^ Weekly cisplatin administered at a dose of 35 to 40 mg/m^2^ is used in some institutions for patients unfit for 100 mg/m^2^ cisplatin every 3 weeks or for patients preferring to receive the treatment in an outpatient setting.

Despite the routine use of 100 mg/m^2^ cisplatin 3-weekly, the optimal dose and timing of cisplatin administration in various chemotherapy protocols have not been elucidated.

The aim of this retrospective analysis was to compare the acute treatment toxicity and its impact on the course of therapy, administered cisplatin and radiation doses, length of hospitalization and supportive drugs administration in patients with HNSCC receiving weekly or 3-weekly cisplatin concurrently with intensity-modulated radiotherapy (IMRT) in a definitive radical upfront setting or after surgical resection in patients with high-risk features in pathology specimen.

## 2 Patients and methods

### 2.1 Data collection

In this retrospective analysis 104 patients with HNSCC were enrolled. The treatment was carried out between September 2013 and September 2014. Radiotherapy was performed in the Department of Radiotherapy in Greater Poland Cancer Center located in Poznan. Chemotherapy was administered in the Department of Medical Oncology in Malgorzata Medical Center in Srem, Serbia. All of the patients underwent a routine staging procedure consisting of physical examination, chest x-ray, computed tomography (CT), magnetic resonance imaging (MRI) or positron emission tomography–computed tomography (PET-CT) scan. Routine laboratory tests such as complete blood count (CBC) and chemistry were performed.

Medical charts were reviewed systematically considering demographics and clinical characteristics including age, sex, gender, weight, body surface area, performance status, comorbidities, stage of the disease and tumor location. Stage was defined based on the American Joint Committee on Cancer (AJCC) TNM version 7 classification. Performance status was evaluated using the Eastern Cooperative Oncology Group (ECOG) scale. Patients with unresectable, locally, and/or regionally advanced disease received radical chemoradiotherapy upfront. Patients after surgical resection for high risk HNSCC received chemoradiotherapy as an adjuvant therapy. Patients with bulky disease received neoadjuvant chemotherapy prior to radical chemoradiotherapy. This retrospective study according to Polish law does not require bioethics committee consent.

### 2.2 Chemotherapy

Fifty patients were treated with 100 mg/m^2^ cisplatin administered 3-weekly (days 1, 22, 43) during radiotherapy. The total planned dose of cisplatin was 300 mg/m^2^. Patients received hydration pre- and post cisplatin administration with 1 L of 0.9% NaCl plus 100 mL of 20% mannitol given before chemotherapy.

Fifty-four patients were treated with weekly 35 mg/m^2^ or 40 mg/m^2^ cisplatin (days 1, 8, 15, 22, 29, 36) during radiotherapy. The total planned dose of cisplatin was 210 to 240 mg/m^2^. Hydration of 1 L 0.9% NaCl was given pre- and postchemotherapy. Additionally, 100 mL of 20% mannitol was given before cisplatin administration.

Neoadjuvant chemotherapy with docetaxel, cisplatin and 5-fluorouracyl (TPF) or cisplatin and 5-fluorouracyl (PF) were administered in 6 patients prior to chemoradiotherapy. Patients unfit for TPF received PF schedule. One patient in the group treated with 100 mg/m^2^ cisplatin received induction chemotherapy with TPF. In the group receiving 35/40 mg/m^2^ cisplatin, 1 patient received TPF and 4 patients PF schedule.

### 2.3 Radiotherapy

All patients received radiotherapy given via an IMRT technique. Radiotherapy was performed with conventional fractionation with a sequential boost. The total planned dose administered to the planning target volume (PTV) in nonresected tumor receiving radiotherapy upfront was 70 Gy. Patients treated in the adjuvant setting received maximum 66 Gy. Radiotherapy was performed 5 days a week.

### 2.4 Supportive medications

In case of coexistent bacterial infection especially in the mucosa of irradiated region, or febrile neutropenia, antibiotics and/or antifungal drugs were administered empirically or according to the specimen culture. Antibiotics used in the study: amoxicillin and clavulanic acid (orally—*po*, intravenous—*iv*), meropenem (*iv*), imipenem (*iv*), ceftriaxone (*iv*), amikacin (*iv*), vancomycin (*iv*), ciprofloxacin (*po, iv*), cefepime (*iv*). Antifungal drugs: nystatin (topical), fluconazole (*po, iv*). In case of severe pain, opioids such as tramadol (*po*), oxycodone (*po*), morphine (*po*, subcutaneous – *sc*), buprenorphine (transdermal—*td*), or fentanyl (*td*) were used.

### 2.5 Toxicity assessment

Treatment-related adverse effects were assessed using National Cancer Institute Common Toxicity Criteria (NCI-CTC) version 4.

### 2.6 Statistical analysis

The comparison of interval data was performed using *t*-student test. The assumption whether data follows normal distribution was checked using Shapiro–Wilks test. The homogeneity of variances was checked with the use of Levene's test. In case data did not follow a normal distribution, non-parametric Mann–Whitney test was used as an alternative.

Nominal data were compared by chi-square test of independence. The percentages of particular events between group A and group B were compared by test of proportions.

Statistical analysis was performed with Statistica 12 (Statsoft, Inc.) software.

All tests were considered significant when *P* < .05.

## 3 Results

### 3.1 Patients characteristics

The patients’ clinical and demographic characteristics are summarized in Table 1. Fifty patients were treated with 100 mg/m^2^ cisplatin 3-weekly concurrently with radiotherapy (group A). Fifty-four patients received radiotherapy and 35 or 40 mg/m^2^ cisplatin weekly (group B). The treatment groups were equally distributed. No significant difference in age, gender, chronic diseases, ECOG performance status, history of cigarette smoking, stage, and localization of primary site were observed between the study arms.

**Table 1:**
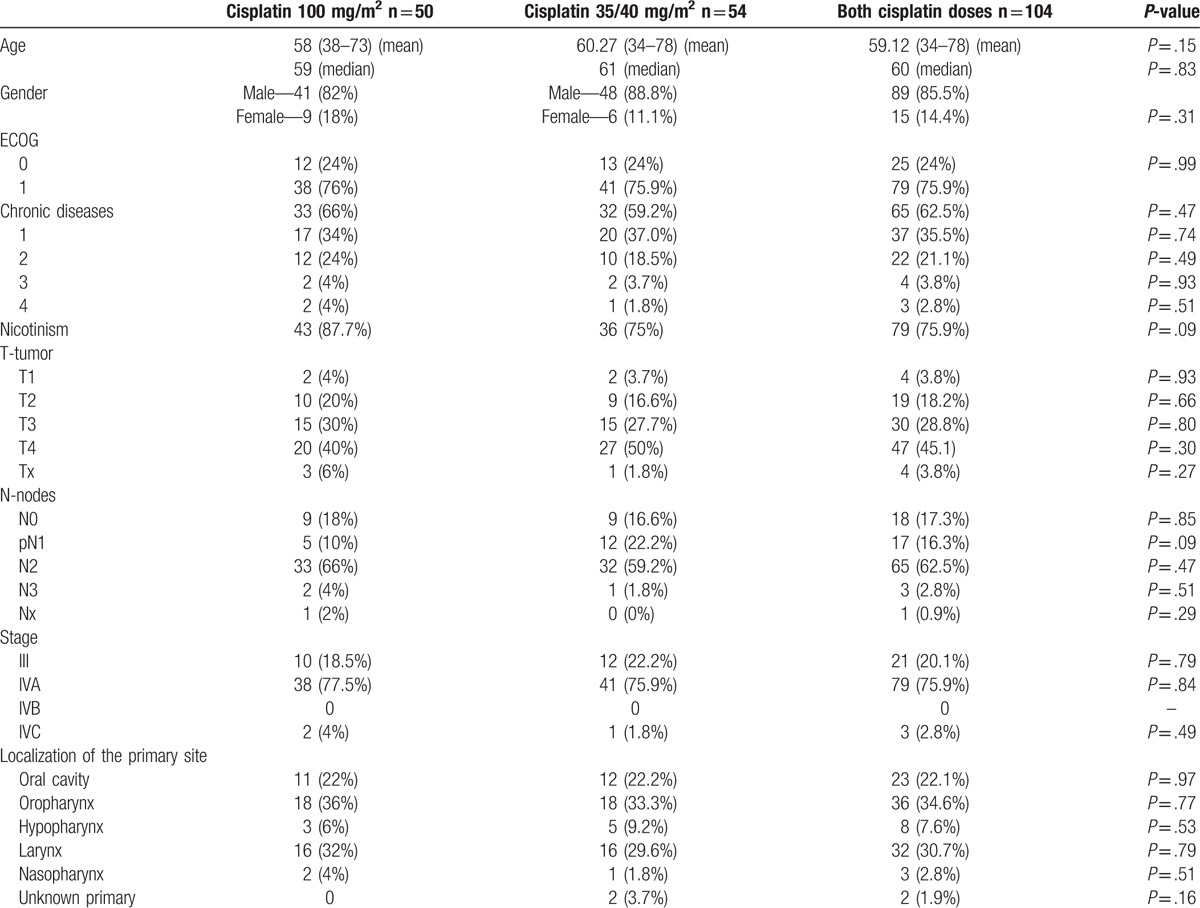
Patients characteristics.

### 3.2 Course of the treatment

Most of the patients were treated with radical chemoradiotherapy upfront (71.1%). Adjuvant chemoradiotherapy after surgical resection was performed in 28.8% of patients. Induction chemotherapy prior to definitive chemoradiotherapy was administered in 5.7% of patients (Table 2).

**Table 2:**
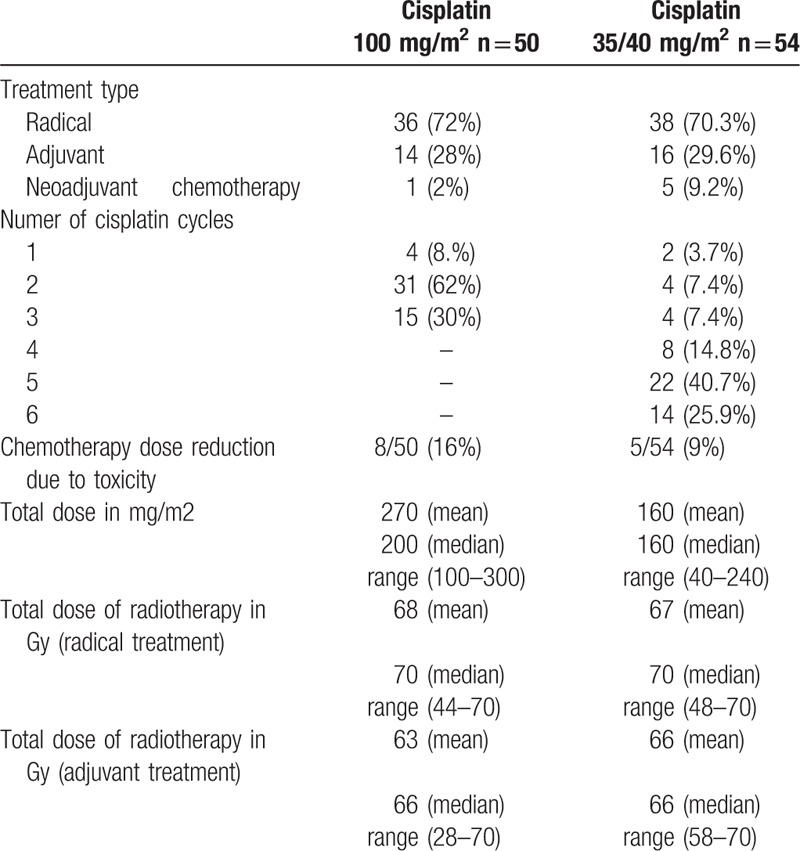
Treatment characteristics.

All patient included in the study were hospitalized from the first day of chemoradiotherapy. The median treatment duration was identical in both studied groups amounted to 46 days (15–56 days). The median duration of hospitalization was independent of cisplatin schedule and was equal to 47 days (16–78 days) in both treatment arms.

### 3.3 Cisplatin and radiotherapy doses

Only in 30% of patients treated with cisplatin 100 mg/m^2^ 3-weekly group who received 3 planned cycles of chemotherapy, dose reduction was necessary in 16% of patients due to adverse events. On the other hand, in the group receiving cisplatin 35/40 mg weekly, 25.9% patients received 6 planned treatment cycles, while dose reduction was performed in 9% of patients. The median dose received in the group treated with cisplatin 100 mg/m^2^ 3-weekly was 200 mg/m^2^ (mean 270 mg/m^2^) and 160 mg/m^2^ (mean 160 mg/m^2^) in those treated with cisplatin 35/40 mg weekly. There was no difference in median and mean dose of radiotherapy delivered in both study arms in patients treated in the definitive and adjuvant setting (Table 2).

The postponement of radiotherapy due to adverse effects was significantly more frequent in patients treated with 35/40 mg/m^2^ of cisplatin in comparison to 100 mg/m^2^ of cisplatin (55.56% vs 32%; *P = *.015). However, the median time of radiotherapy postponement was only 1 day (mean 1.74; [0–10]) in group B and 0 days (mean 1.18; [0–8]) in group A.

### 3.4 Toxicity

**Figure 1:**
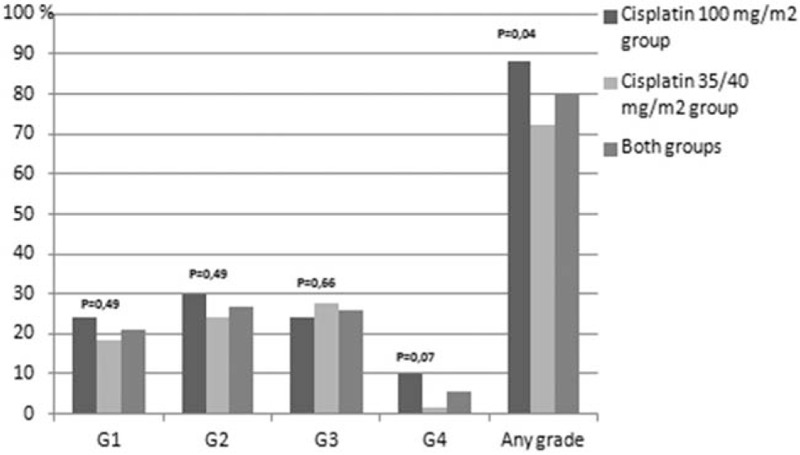
Hematological toxicity leukopenia.

**Figure 2:**
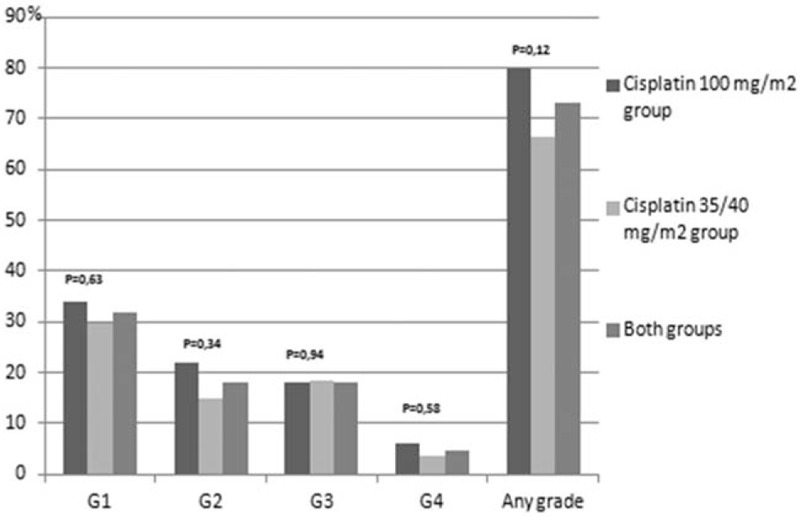
Hematological toxicity neutrophilopenia.

**Figure 3:**
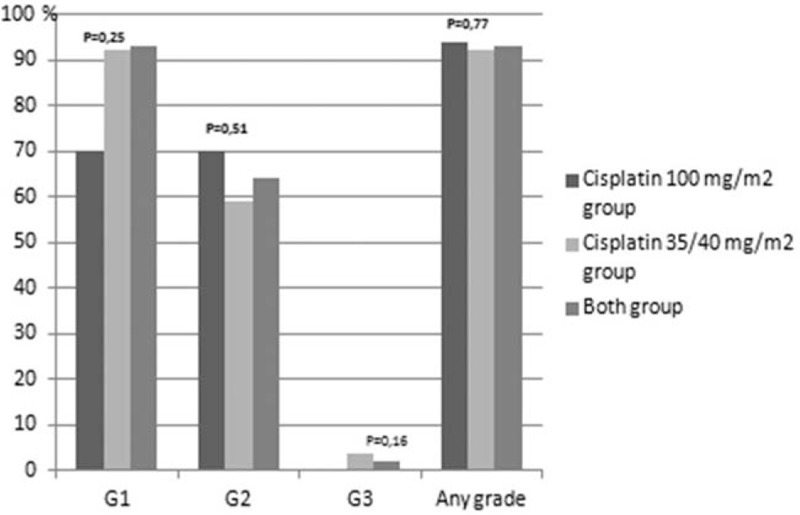
Hematological toxicity anemia.

**Figure 4:**
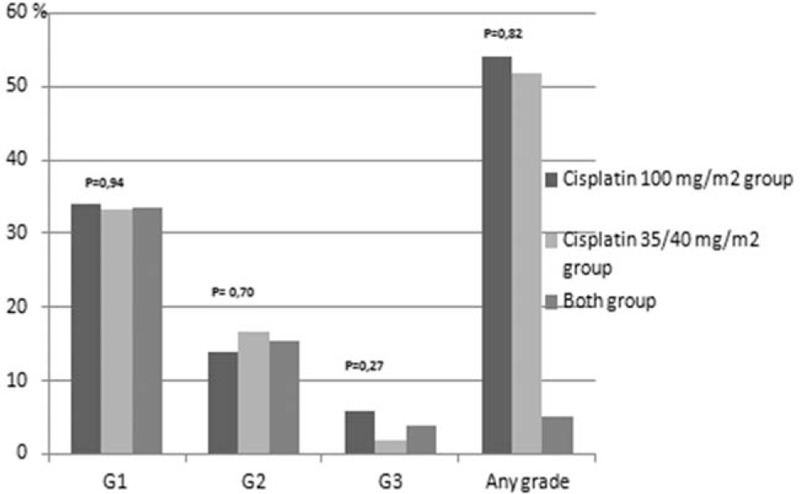
Hematological toxicity thrombocytopenia.

**Figure 5:**
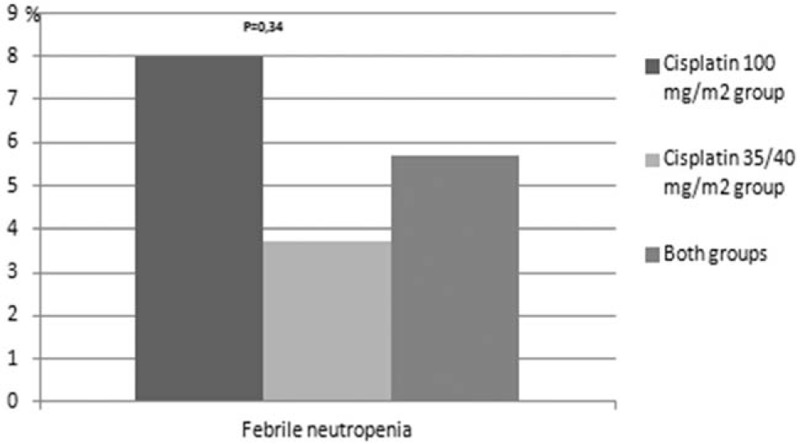
Hematological toxicity febrile neutropenia.

**Figure 6:**
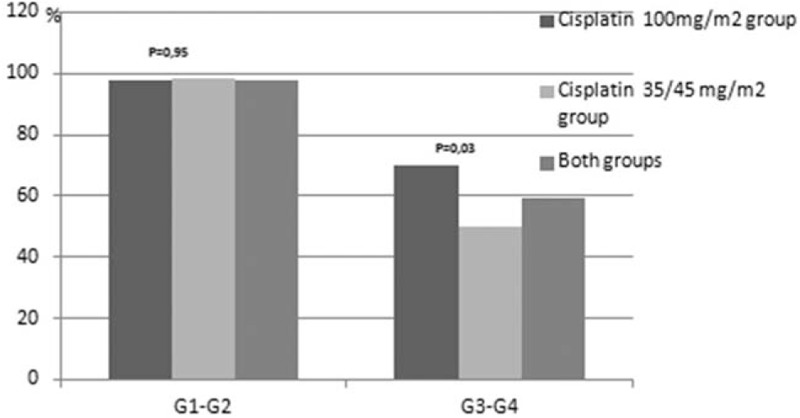
Mucositis.

**Figure 7:**
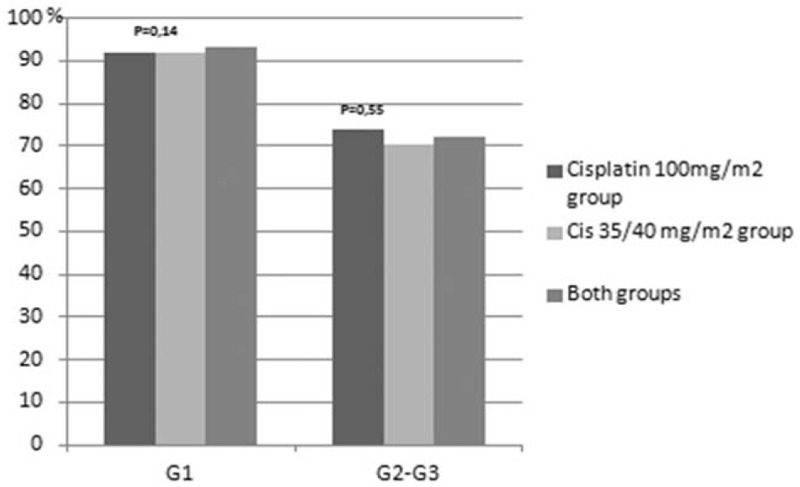
Skin toxicity.

**Figure 8:**
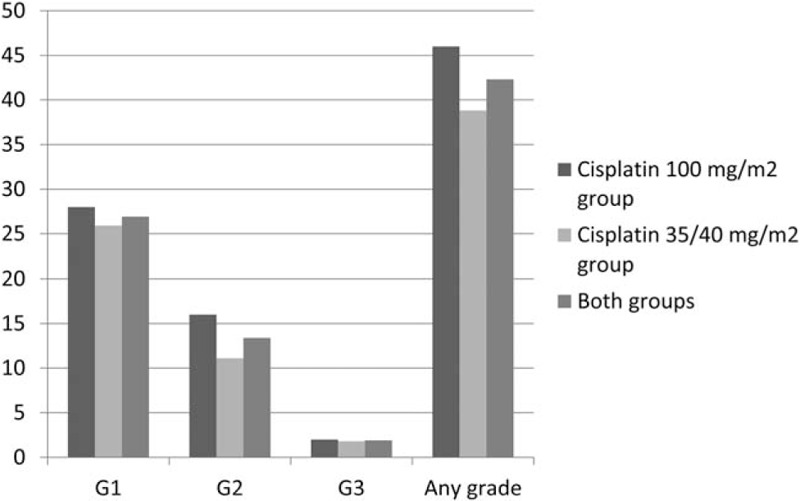
Kidney injury. Acute kidney injury in patients treated with chemoradiotherapy cisplatin 100 mg/m2 group, 35/40 mg/m2 group and both cisplatin groups.

Treatment related toxicities are presented in Figures 1–8. Radiotherapy administered with 100 mg/m^2^ cisplatin 3-weekly was linked with statistically significant increase in leukopenia (any grade) in comparison to 35/40 mg/m^2^ of cisplatin weekly—Figure 1; (88% vs 72.2%; *P = *.04). Also grade 4 leukopenia occurred more frequently in patients receiving 3-weekly cisplatin. Febrile neutropenia occurred more often in this group (Fig. 5), however this difference was not statistically significant (8% vs 3.7%; *P = *.34).

Grade 3 and 4 mucositis occurred more frequently in patients treated in group A (70% vs 50%; *P = *.037). The median time of developing grade 3/4 mucositis was 33 days in both groups (Fig. 6).

There was no difference in acute kidney injury incidence (group A: 46% vs group B: 38.89%; *P = *.46) and severity between the studied groups (Fig. 8). The median onset time to acute kidney failure was 16 (mean 15.8) days (group A) and 18 (mean 23) days (group B). Most of them resolved completely after hydration (group A: 36% vs group B: 25.93%; *P = *.26). None of the patients required renal replacement therapy.

Regardless of the prophylactic administration of antiemetic drugs (ondansetron and dexamethasone) nausea and vomiting occurred in 70% of patients treated with 3-weekly cisplatin and 51.85% of patients receiving weekly cisplatin (*P = *.058).

Hypo- or hyperthyroidism was observed in 22.22% of patients in group A and 29.73% treated in group B (*P = *.5)

Other adverse events like fatigue, electrolyte abnormalities, diarrhea, constipation, elevated aminotransferases, hyperbilirubinemia or deep vein thrombosis were noted in 32% of patients in group A and 31.48% treated in group B (*P = *.95).

Mortality during treatment occurred in 10 (9.6%) patients (group A: 12% vs group B: 7.41%; *P = *.42). Death occurred due to complication of stroke, sepsis, hemorrhage from the primary tumor or pulmonary edema.

### 3.5 Supportive medication during chemoradiotherapy

Antibiotics administered due to infection or febrile neutropenia were more frequently applied in patients treated in group A (85.19%) in comparison to group B (76%), however the difference was not statistically significant (*P = *.23).

In all treated patients the median time to antibiotics administration calculated from the first day of hospitalization was 24.5 days (group A: 26 days vs group B: 24 days; *P = *.64). The median time of antibiotics use was 10 days (group A: 10 days vs group B: 11 days; *P = *.059).

The frequency of topically applied antifungal drugs due to oral cavity mycosis or prevention of its occurrence was similar in both groups (A: 94% vs B: 90.74%; *P = *.53).

In all treated patients the median time to antifungal drugs administration calculated from the first day of hospitalization was 13 days (group A: 14 days vs group B: 13 days; *P = *.64). The median time of antifungal drugs use was 35 days (group A: 35 days vs group B: 34 days; *P = *.51).

The frequency of opioids used due to cancer related or treatment related pain (skin and mucous membrane radiation reactions) was similar in both study groups.

Tramadol was administered in 74% (group A) and 79.63% (group B) of patients (*P = *.49). In all treated patients the median time to tramadol administration calculated from the first day of hospitalization was 9 days (group A: 8 days vs group B: 9.5 days; *P = *.97).

Strong opioids like oxycodone, morphine, buprenorphine or fentanyl were administered with a similar frequency in both study groups (group A: 70% vs group B 73.58%; *P = *.68).

In all treated patients the median time to strong opioids administration calculated from the first day of hospitalization was 17 days (group A: 18 days vs group B: 15 days; *P = *.64).

## 4 Discussion

Most of the randomized controlled trials have accepted cisplatin in a dose of 100 mg/m^2^ administered every 3 weeks concurrently with radiation as a standard reference regimen in HNSCC patients treated in the definitive and adjuvant setting. However, high toxicity, treatment compliance and additional supportive care lead to modification of this chemotherapy scheme. Moreover, suboptimal compliance with 100 mg/m^2^ cisplatin can influence the treatment outcome, resulting in shorter survival.^[12–14]^ Also the intent of cisplatin scheduling modifications was to increase the cumulative cisplatin dose and consequently the efficacy of combined therapy. Another rationale for administration of lower doses of cisplatin in shorter intervals was to provide radio-sensitizing chemotherapy during a larger proportion of the courses of radiotherapy.^[15]^

The administration of planned chemotherapy dose is very important for the improvement of cancer patients’ survival. A recently presented systematic review including 6 definitive chemoradiotherapy phase 3 trials, demonstrated statistically significant association between cumulative cisplatin dose (independent of the schedule) and overall survival benefit observed for higher doses. A 2.2% absolute benefit in OS between the chemoradiotherapy and radiotherapy arm was observed for every 10 mg increase in the cumulative cisplatin dose (range of used cisplatin doses: 140–270 mg/m^2^).^[16]^ In our study patients treated with weekly cisplatin received lower median cumulative dose of cisplatin, which might have been linked to worse treatment outcomes in this group of patients. Similarly in other studies patients in the weekly cisplatin group received lower median cumulative dose than those in 3-weekly arm.^[17,18]^ This finding can, to a certain extent, be explained by the reduced compliance of some patients refusing the administration of last or 2 last doses of weekly cisplatin. Moreover, in our study postponement of radiotherapy due to adverse events was more frequent in patients treated with weekly cisplatin. However, the median time of radiotherapy postponement was only 1 day, which probably did not affect treatment efficacy. Mucositis is one of the most common dose-limiting toxicity in both cisplatin dosing schemes (3-weekly and weekly). In our study we observed higher incidence of grade 3 to 4 mucositis in patients receiving 100 mg/m^2^ 3-weekly than 35/40 mg/m^2^ weekly cisplatin (70% vs 50%; *P = *.037). The so far published results are very inconsistent. Some studies demonstrated higher incidence of grade 3 to 4 mucositis in patients treated in the 3-weekly cisplatin scheme.^[19]^ Other do not show any difference, some demonstrate higher frequency in the weekly cisplatin schedule or a trend towards higher incidence.^[20,21,17]^ Our results are consistent with the currently largest study presented by Fayette and colleagues.

One of the arguments for the fractionated doses of cisplatin administered with radiation was higher incidence of grade 3 to 4 neutropenia occurring in about 30% of patients treated with 3-weekly cisplatin schedule compared to 10–15% observed in weekly cisplatin administration.^[5,22–26]^ In our study, we did not observe any difference in the incidence of grade 3 to 4 neutropenia. Our data was consistent with other studies demonstrating no difference in hematological toxicities between the 2 comparative arms.^[20,21]^ However, we observed a significant difference in grade 1 to 4 leukopenia between both arms (88%, 3-weekly vs 72.2%, weekly cisplatin; *P = *.04), with a trend towards higher incidence in grade 4 leukopenia in patients receiving 3-weekly cisplatin (10% vs 1.85%; *P = *.07). Grade 4 leukopenia was linked to low lymphocyte count.

Cisplatin is a cytotoxic agent causing nephrotoxicity. Most of the chemoradiotherapy studies conducted in HNSCC demonstrated higher incidence of renal toxicity with 3-weekly than weekly cisplatin.^[18,19,25]^ Other studies did not demonstrate any difference.^[27]^ In our study there was no difference in the acute kidney failure, however there was a trend towards higher incidence in the group receiving 3-weekly cisplatin than weekly (46% vs 38.89%; *P = *.46). Grade 3 acute nephrotoxicity was observed only in 1 patient in each study group. Our results are consistent with another study reporting no statistically significant difference between study arms with a trend towards higher frequency of nephrotoxicity in 3-weekly scheme.^[17]^

To our knowledge this is the first study comparing the use of supportive treatment in patients with HNSCC receiving 3-weekly or weekly cisplatin schedule concurrent with radiotherapy. About 80% of patients receiving chemoradiotherapy required antibiotic administration. In our study, we observed no statistically significant difference in the frequency of antibiotics use between studied groups. However, there was a trend towards more frequent application of antibiotics in patients receiving 100 mg/m^2^ of cisplatin (85.19% vs 76%; *P = *.23). Also in this group, we observed a trend towards a longer median time of antibiotic intake (16.5 vs 12.39 days; *P = *.08). This observation is linked mainly to a higher frequency of grade 3 or 4 mucositis, skin toxicity and febrile neutropenia in this group.

Treatment related to skin toxicity and mucositis in patients with HNSCC receiving chemoradiotherapy is often related to pain of the radiated area. Pain intensity increases with the severity of these adverse effects. Initially, most of the patients require weak opioids application with subsequent administration of strong opioids when toxicity is higher. Most of the patients require dose escalation of strong opioids during the course of the treatment. The majority of patients participating in our study required tramadol (76.92%) and subsequently strong opioids (71.15%). Most patients required weak opioids application just after the onset of low-grade skin toxicity (median 14 days) and mucositis (12 days). The onset of high-grade skin toxicity and mucositis occurred at a median time of 33 days, and was linked to dose escalation of strong opioids. In some patients, pain was a symptom of cancer occurrence and opioids were also administered in this situation. The median time to tramadol and strong opioids administration was 9 and 17 days respectively. There was no statistically significant difference in time to antibiotics, tramadol or strong opioids administration between studied groups. Also, the study did not show any differences between the study arms in the number of patients taking tramadol and strong opioids. This analysis is very important since antibiotics used during chemoradiotherapy might increase treatment toxicity (e.g., nephrotoxicity, ototoxicity, hematological toxicity). Moreover, antibiotics or opioids might interact with other prescribed drugs causing further toxicity. All this might influence the course of the treatment and subsequently patients’ prognosis. These results demonstrated that the selection of chemotherapy schedule (weekly or 3-weekly cisplatin) does not have any influence on frequency of antibiotics or opioids administration and does not affect the duration of treatment with these drugs.

The limitation of this study is the lack of data supporting the efficacy of these 2 treatments. Currently, it is not clear whether radiation delivered with 30 to 40 mg/m^2^ of cisplatin weekly is as effective as the standard chemoradiotherapy scheme with 100 mg/m^2^ of cisplatin administered 3-weekly in patients with HNSCC. The efficacy of weekly (30–40 mg/m^2^) in comparison to 3-weekly (100 mg/m^2^) cisplatin schedule delivered with radiation was evaluated in 5 retrospective and 1 randomized study. These studies demonstrated inconsistent results in terms of treatment outcomes. In 2 adjuvant studies: 1 small, randomized and 1 retrospective, there was no significant difference in survival between the studied groups.^[21,28]^ Other retrospective study of patients receiving definitive or adjuvant chemoradiotherapy suggested that 100 mg/m^2^ of cisplatin resulted in better OS (overall survival) and similar PFS (progression-free survival) compared to weekly cisplatin. However, patients receiving weekly cisplatin were significantly older, which likely had introduced a bias.^[17]^ In another study conducted on a similar group of patients, 100 mg/m^2^ of cisplatin resulted in longer PFS and OS on univariate analysis but not on multivariate analysis.^[19]^ Another similar study did not demonstrate a difference in survival between the studied groups.^[20]^ The most recent study on patients with locally advanced HNSCC receiving definitive chemoradiotherapy demonstrated improvement in locoregional control and OS when cisplatin was delivered 3-weekly in comparison to the weekly scheme.^[29]^

The main advantage of our study is an equally distributed and randomized study arms in terms of age, gender, ECOG performance status, chronic diseases, stage of the disease and localization of primary tumor. Other studies included weekly administration of cisplatin to unfit patients and factors such as age, weight loss, kidney failure, performance status and the use of induction chemotherapy were taken into consideration for choosing weekly or 3-weekly cisplatin schedule, which might have introduced a bias.^[17,19]^ Based on our results, the main difference in patients treated with 3-weekly cisplatin is higher incidence of acute severe mucositis in comparison to the weekly cisplatin schedule. In general, there was no significant difference in severe skin toxicity, acute kidney injury or hematological toxicity. Also, the scheme of cisplatin dosing did not determine the duration of hospitalization, quantities of supportive drugs used or total doses of received radiotherapy. However, patients treated with weekly scheme received lower total cisplatin dose in comparison to those treated in the 3-weekly schedule which might have affected the treatment efficacy. The limitation of this study is the fact that the data presenting overall survival has not been presented. In conclusion, our results did not demonstrate the advantage of weekly cisplatin. Therefore, the standard 3-weekly schedule is probably more appropriate for patients with HNSCC, considering the need and importance of close monitoring, mainly in regards to severe mucositis. However, retrospective comparisons of the efficacy, acute toxicity and compliance of weekly and 3-weekly cisplatin schedules reported conflicting results.^[17,18,21,27,28,30,31]^ Most appropriate cisplatin scheme given concurrently with radiation therapy in patients with HNSCC treated in the adjuvant or definitive setting requires further clarification in randomized, controlled studies.

## References

[1] Krzakowski M Potemski P Warzocha K Onkologia Kliniczna. Nowotwory narządów głowy i szyi. VM Media sp z o o, Gdańsk 2013;1–32.

[2] Pignon JP Bourhis J Domenge C Chemotherapy added to locoregional treatment for head and neck squamous-cell carcinoma: three meta-analyses of updated individual data. MACH-NC Collaborative Group. Meta-Analysis of Chemotherapy on Head and Neck Cancer. Lancet 2000;355:949–55. 10768432

[3] Pignon JP le Maıtre A Maillard E MACH-NC Collaborative Group. Meta-analysis of chemotherapy in head and neck cancer (MACHNC): an update on 93 randomised trials and 17,346 patients. Radiother Oncol 2009;92:4–14. 19446902 10.1016/j.radonc.2009.04.014

[4] Gregoire V Lefebvre JL Licitra L EHNS-ESMO-ESTRO Guidelines Working Group. Squamous cell carcinoma of the head and neck: EHNS-ESMO-ESTRO Clinical Practice Guidelines for diagnosis, treatment and follow-up. Ann Oncol 2010;21 (suppl 5):v184–6. 20555077 10.1093/annonc/mdq185

[5] Cooper JS Pajak TF Forastiere AA Postoperative concurrent radiotherapy and chemotherapy for high-risk squamous-cell carcinoma of the head and neck. N Engl J Med 2004;350:1937–44. 15128893 10.1056/NEJMoa032646

[6] Bernier J Domenge C Ozsahin M Postoperative irradiation with or without concomitant chemotherapy for locally advanced head and neck cancer. N Engl J Med 2004;350:1945–52. 15128894 10.1056/NEJMoa032641

[7] Bachaud JM Cohen-Jonathan E Alzieu C Combined postoperative radiotherapy and weekly cisplatin infusion for locally advanced head and neck carcinoma: final report of a randomized trial. Int J Radiat Oncol Biol Phys 1996;36:999–1004. 8985019 10.1016/s0360-3016(96)00430-0

[8] Huguenin P Beer KT Allal A Concomitant cisplatin significantly improves locoregional control in advanced head and neck cancers treated with hyperfractionated radiotherapy. J Clin Oncol 2004;22:4665–73. 15534360 10.1200/JCO.2004.12.193

[9] Jeremic B Shibamoto Y Stanisavljevic B Radiation therapy alone or with concurrent low-dose daily either cisplatin or carboplatin in locally advanced unresectable squamous cell carcinoma of the head and neck: a prospective randomized trial. Radiother Oncol 1997;43:29–37. 9165134 10.1016/s0167-8140(97)00048-0

[10] Rampino M Ricardi U Munoz F Concomitant adjuvant chemoradiotherapy with weekly low-dose cisplatin for high-risk squamous cell carcinoma of the head and neck: a phase II prospective trial. Clin Oncol (R Coll Radiol) 2011;23:134–40. 21030225 10.1016/j.clon.2010.09.004

[11] Sharma A Mohanti BK Thakar A Concomitant chemoradiation versus radical radiotherapy in advanced squamous cell carcinoma of oropharynx and nasopharynx using weekly cisplatin: a phase II randomized trial. Ann Oncol 2010;21:2272–7. 20427350 10.1093/annonc/mdq219

[12] Pajak TF Laramore GE Marcial VA Elapsed treatment days—a critical item for radiotherapy quality control review in head and neck trials: RTOG report. Int J Radiat Oncol Biol Phys 1991;20:13–20. 1993621 10.1016/0360-3016(91)90132-n

[13] Browman GP Hodson DI Mackenzie RG Cancer care ontario practice guideline initiative head and neck cancer disease site group: choosing a concomitant chemotherapy and radiotherapy regimen for squamous cell head and neck cancer: a systematic review of published literature with subgroup analysis. Head Neck 2001;23:579–89. 11400247 10.1002/hed.1081

[14] Adelstein DJ Li Y Adams GL An intergroup phase III comparison of standard radiation therapy and two schedules of concurrent chemoradiotherapy in patients with unresectable squamous cell head and neck cancer. J Clin Oncol 2003;21:92–8. 12506176 10.1200/JCO.2003.01.008

[15] Kurihara N Kubota T Hoshiya Y Pharmacokinetics of cis-diamminedichloroplatinum (II) given as low-dose and high-dose infusions. J Surg Oncol 1996;62:135–8. 8649040 10.1002/(SICI)1096-9098(199606)62:2<135::AID-JSO10>3.0.CO;2-7

[16] Strojan P Vermorken JB Beitler JJ Cumulative cisplatin dose in concurrent chemoradiotherapy for head and neck cancer: a systematic review. Head Neck 2016;38 (suppl 1):E2151–8. 25735803 10.1002/hed.24026

[17] Espeli V Zucca E Ghielmini M Weekly and 3-weekly cisplatin concurrent with intensity-modulated radiotherapy in locally advanced head and neck squamous cell cancer. Oral Oncol 2012;48:266–71. 22079100 10.1016/j.oraloncology.2011.10.005

[18] Rades D Kronemann S Meyners T Comparison of four cisplatin-based radiochemotherapy regimens for nonmetastatic stage III/IV squamous cell carcinoma of the head and neck. Int J Radiat Oncol Biol Phys 2011;80:1037–44. 20638185 10.1016/j.ijrobp.2010.03.033

[19] Fayette J Molin Y Lavergne E Radiotherapy potentiation with weekly cisplatin compared to standard every 3 weeks cisplatin chemotherapy for locoregionally advanced head and neck squamous cell carcinoma. Drug Des Devel Ther 2015;26:6203–10. 10.2147/DDDT.S81488 PMC4664534 26648696

[20] Kose F Besen A Sumbul T Weekly cisplatin versus standard 3-weekly cisplatin in concurrent chemoradiotherapy of head and neck cancer: the Baskent University Experience. Asian Pac J Cancer Prev 2011;12:1185–8. 21875263

[21] Tsan DL Lin CY Kang CJ The comparison between weekly and 3-weekly cisplatin delivered concurrently with radiotherapy for patients with postoperative high-risk squamous cell carcinoma of the oral cavity. Radiat Oncol 2012;7:215. 23245290 10.1186/1748-717X-7-215 PMC3564896

[22] Zenda S Onozawa Y Tahara M Feasibility study of single agent cisplatin and concurrent radiotherapy in Japanese patients with squamous cell carcinoma of the head and neck: preliminary results. Jpn J Clin Oncol 2007;37:725–9. 17925299 10.1093/jjco/hym106

[23] Homma A Inamura N Oridate N Concomitant weekly cisplatin and radiotherapy for head and neck cancer. Jpn J Clin Oncol 2011;41:980–6. 21715362 10.1093/jjco/hyr086

[24] Mitra D Choudhury K Rashid MA Concurrent chemotherapy in advanced head and neck carcinoma—a prospective randomized trial. Bangladesh J Otorhinolaryngol 2011;17:88–95.

[25] Kiyota N Tahara M Okano S Phase II feasibility trial of adjuvant chemoradiotherapy with 3-weekly cisplatin for Japanese patients with post-operative high-risk squamous cell carcinoma of the head and neck. Jpn J Clin Oncol 2012;42:927–33. 22923484 10.1093/jjco/hys128

[26] Osman N Elamin YY Rafee S Weekly cisplatin concurrently with radiotherapy in head and neck squamous cell cancer: a retrospective analysis of a tertiary institute experience. Eur Arch Otorhinolaryngol 2014;271:2253–9. 24121822 10.1007/s00405-013-2749-9

[27] Ho KF Swindell R Brammer CV Dose intensity comparison between weekly and 3-weekly cisplatin delivered concurrently with radical radiotherapy for head and neck cancer: a retrospective comparison from New Cross Hospital, Wolverhampton, UK. Acta Oncol 2008;47:1513–8. 18607863 10.1080/02841860701846160

[28] Geiger JL Lazim AF Walsh FJ Adjuvant chemoradiation therapy with high-dose versus weekly cisplatin for resected, locally-advanced HPV/p16-positive and negative head and neck squamous cell carcinoma. Oral Oncol 2014;50:311–8. 24467937 10.1016/j.oraloncology.2014.01.001

[29] Rades D Seidl D Janssen S Comparison of weekly administration of cisplatin versus three courses of cisplatin 100 mg/m(2) for definitive radiochemotherapy of locally advanced head-and-neck cancers. BMC Cancer 2016;8:437. 10.1186/s12885-016-2478-8 PMC4938984 27391309

[30] Geeta SN Padmanabhan TK Samuel J Comparison of acute toxicities of two chemotherapy schedules for head and neck cancers. J Cancer Res Ther 2006;2:100–4. 17998687 10.4103/0973-1482.27584

[31] Uygun K Bilici A Karagol H The comparison of weekly and three-weekly cisplatin chemotherapy concurrent with radiotherapy in patients with previously untreated inoperable non-metastatic squamous cell carcinoma of the head and neck. Cancer Chemother Pharmacol 2009;64:601–5. 19123002 10.1007/s00280-008-0911-7

